# Metformin Inhibits Mouse Islet Insulin Secretion and Alters Intracellular Calcium in a Concentration-Dependent and Duration-Dependent Manner near the Circulating Range

**DOI:** 10.1155/2018/9163052

**Published:** 2018-03-18

**Authors:** Lindor Gelin, Jiewen Li, Kathryn L. Corbin, Ishrat Jahan, Craig S. Nunemaker

**Affiliations:** ^1^Department of Biomedical Sciences, Heritage College of Osteopathic Medicine, Ohio University, Athens, OH, USA; ^2^Diabetes Institute, Heritage College of Osteopathic Medicine, Ohio University, Athens, OH, USA

## Abstract

Metformin is considered the first-line treatment for type 2 diabetes. While metformin primarily increases insulin sensitivity, evidence also suggests that metformin affects the activity of insulin-secreting pancreatic islets. This study was designed to systematically examine the direct effects of metformin by measuring insulin secretion and the kinetics of the calcium response to glucose stimulation in isolated mouse islets using varying concentrations (20 *μ*M, 200 *μ*M, and 1 mM) and durations (~1, 2, and 3 days) of metformin exposure. We observed both concentration- and duration-dependent inhibitory effects of metformin. Concentrations as little as 20 *μ*M (nearing circulating therapeutic levels) were sufficient to reduce insulin secretion following 3-day treatment. Concentrations of 200 *μ*M and 1 mM produced more pronounced effects more rapidly. With 1 mM metformin, islets showed severe impairments in calcium handling, inhibition of insulin secretion, and increased cell death. No stimulatory effects of metformin were observed for any experimental endpoint. We conclude that the direct effects of metformin on islets are inhibitory at near-physiological concentrations. Beneficial effects of metformin observed on islets under various stressors may occur by “resting” fatigued cellular processes. However, metformin may have unintended consequences on normally functioning islets within the circulating range that require further evaluation.

## 1. Introduction

The first line of pharmacological treatment for patients with type 2 diabetes (T2D) is metformin [1]. Metformin is thought to reduce insulin resistance in diabetic patients primarily by inhibiting the mitochondrial respiratory-chain complex in hepatocytes to decrease hepatic glucose production, thus leading to a decrease in blood glucose concentrations [2, 3]. In addition, metformin has effects on the gut including increased intestinal glucose uptake and increased GLP-1 levels, as well as possible effects on the gut microbiome [4, 5].

Although the mechanism of action for metformin is fairly well understood in hepatocytes, the direct effects metformin has on pancreatic islets and beta cells are less clear. In conditions of stress, metformin treatment appears to ameliorate the effects of diabetes on islets by reducing or inhibiting damaging levels of activity related to hyperglycemia and deranged lipids [6], nitric oxide signaling [7], lipotoxic endoplasmic reticulum stress [8], and oxidative stress [9]. Metformin has shown numerous beneficial effects in islets isolated from T2D patients including an increase in insulin granules, insulin mRNA expression, and glucose-stimulated insulin secretion but also a reduction in both apoptosis and oxidative stress [10]. There is evidence that metformin affects glucagon-secreting alpha cells in the islet as well [11]. In all of these studies, unhealthy islets/beta cells appear to benefit from inhibitory or restful effects of metformin to relieve stress.

While there is an apparent restorative and protective effect of metformin on beta cells in a stressed environment, there is relatively little evidence of stimulatory effects of metformin on insulin secretion [12]. Biguanides including metformin as a general rule do not appear to stimulate insulin secretion [13] but can have inhibitory effects [14]. In both clonal and human islets, metformin has been shown to inhibit glucose-stimulated insulin secretion (GSIS) by increasing AMP-activated protein kinase activity [15]. However, typical circulating levels [16–19] of metformin in the body are 1-2% of the concentrations used in these studies or lower.

The focus of the present study was to determine the effects of metformin on normal rodent pancreatic islets in a systematic concentration- and time-dependent manner. Measuring secreted insulin accumulation in media, glucose-stimulated insulin secretion, and glucose-stimulated [Ca^2+^]_i_ responses, we demonstrate a clear inhibitory effect of metformin that increases with increasing concentration and/or duration. Importantly, these in vitro effects of metformin are observed at concentrations surprisingly close to the therapeutic range of circulating levels found in blood [16, 18]. These findings provide a possible caveat to the use of metformin in individuals with normally functioning islets.

## 2. Materials and Methods

### 2.1. Mice

Studies were conducted using outbred male CD-1 mice at ages of 8–12 weeks (Envigo, Indianapolis, IN). All animal procedures were approved by the Ohio University Institutional Animal Care and Use Committee.

### 2.2. Islet Isolation and Dispersion

Pancreatic islets were isolated by collagenase-P digestion (Roche Diagnostics, Indianapolis, IN) followed by centrifugation with Histopaque 1100 (Sigma-Aldrich, St. Louis, MO) as previously described [20]. Islets were incubated overnight in RPMI 1640 medium containing 11 mM glucose (Invitrogen) supplemented with 10% fetal bovine serum and 1% penicillin/streptomycin to allow recovery from collagenase digestion before further treatment.

### 2.3. [Ca^2+^]_i_ Imaging

Fura-2 AM fluorescence imaging was utilized to measure [Ca^2+^]_i_ levels as previously described [21]. Briefly, perifused solutions first passed through an inline heater to a temperature of 35+/−3 degrees Celsius into an open diamond bath imaging chamber (Warner Instruments, Cat: 64-0288) which was mounted using a stage adapter (Warner Instruments, Cat: 64-0298). Observation of islets was performed using a Hamamatsu ORCA-Flash4.0 digital camera (Hamamatsu Photonics K.K., Hamamatsu City, Japan, Model C11440-22CU) mounted on a BX51WIF fluorescence microscope with a 10x objective (Olympus, Tokyo, Japan). Excitation light was provided by a xenon burner supplied to the image field through a light pipe and filter wheel (Sutter Instrument Co., Novato CA, Model LB-LS/30) with a Lambda 10-3 Optical Controller (Sutter Instrument Co., Novato, CA, Model LB10-3-1572). Images were taken sequentially with 340 nm and 380 nm excitation to produce each [Ca^2+^]_i_ ratio from emitted light at 510 nm. Data were analyzed using cellSens Dimension 1.13 imaging software (Olympus, Tokyo, Japan).

For [Ca^2+^]_i_ imaging, all islets were loaded with 1 *μ*M fura-2 AM and CellTracker Red was also included during fura-2 loading for one of the two treatment groups in order to pair together and simultaneously image islets from untreated and metformin-pretreated groups as published previously [22, 23]. By recording control and test groups simultaneously, this approach controls for temperature, perifusion rate, and other variables, which allows us to identify subtle changes in islet function including signs of endoplasmic reticulum (ER) stress by examining the latency, amplitude, and slope of islet [Ca^2+^]_i_ responses to glucose stimulation [22–27].

### 2.4. Insulin Secretion

Sets of 20 islets/well in 12-well or 24-well plates were used for all studies of insulin secretion as previously described [25, 28]. Media was collected at ~24 h intervals or during glucose-stimulated insulin secretion (GSIS) tests using 3 mM and 28 mM glucose stimulation. Insulin secretion was measured by standard insulin enzyme-linked immunosorbent assay (ELISA) following manufacturer's directions (ALPCO, Salem, NH). Intra-assay variability was kept to below 15% for all studies.

### 2.5. Cell Death Measurements

Apoptosis was measured using annexin V (488 nm excitation/525 nm emission), which detects phosphatidylserine when it is exposed to the outer leaflet of the plasma membrane during apoptosis. Propidium iodide (535 nm excitation/620 nm emission), which is a cell exclusion dye, was used to detect generalized cell death. Regions of interest were drawn around islets to measure fluorescence intensity per islet for each individual islet.

### 2.6. Statistical Analysis

**Figure 1:**
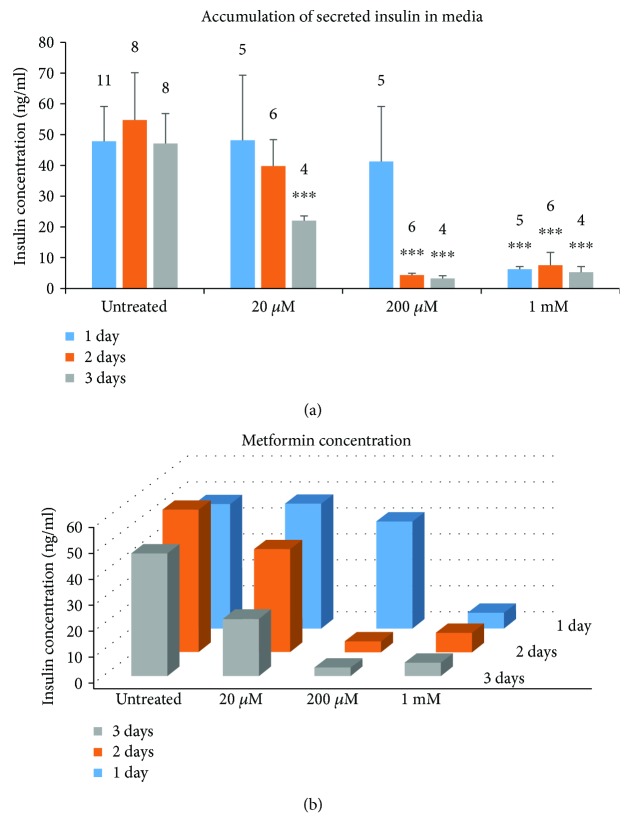
Metformin exposure reduces insulin accumulation in media in a concentration- and duration-dependent manner. Media were collected from islets exposed to varying concentrations of metformin at 1-day intervals following 1, 2, or 3 days of exposure conducted in three separate trials. Insulin accumulation during these time periods was measured by ELISA. (a) Insulin secretion (Mean +/− SEM) for each concentration of metformin on the *x*-axis is displayed sequentially over time. (b) 3D plot of the same data to display both the time-dependent (back-to-front) and concentration-dependent (left-to-right) effects of metformin. Sets of 20 size-matched islets were used for each replicate. Numbers above each column indicate the number of replicates. ^∗∗∗^*P* < 0.001.

Unless otherwise stated, a Student *t*-test was used to compare untreated to metformin-treated islets for each set of experiments. We additionally performed a Benjamini-Hochberg multiple comparison correction to the key findings displayed in Figure 1 and found the data to remain significant to a *P* value <0.001 for all significant differences originally reported. Outlier data points were removed from calcium data using a cutoff of 2 times the standard deviation of the mean. High/low points were removed from insulin accumulation studies as described in the Results. A 2 × 2 contingency table and Fisher's exact test were used to determine differences in oscillatory capacity between metformin-treated and untreated islets.

## 3. Results and Discussion

### 3.1. Metformin Effects on Insulin Accumulation in Media

We first examined insulin release that accumulated into RPMI media containing 11 mM glucose during treatment with varying concentrations of metformin in islets isolated from outbred CD-1 mice. Media was collected daily to measure insulin at daily intervals. Islets were given fresh media daily and retreated with fresh metformin. Insulin accumulation varied widely over the ~24 h sampling periods, so the highest and lowest insulin value from each treatment group was removed to decrease variability. As shown in Figure 1(a), the insulin accumulation did not change from day to day in the untreated control group (see first set of three columns). Islets treated with 20 *μ*M metformin produced a time-dependent decrease over 1-, 2-, and 3-day incubation, with a significant ~50% inhibition at day 3. In the 200 *μ*M metformin treatment group, there was a small decrease in the day 1 group and significant decreases in the day 2 and day 3 groups in comparison to the untreated groups. All of the 1 mM metformin treatment groups showed a powerful and significant decrease in insulin accumulation when compared to the untreated groups for each incubation period. In Figure 1(b), the same data are plotted to show both the concentration-dependent effects (left to right) and time-dependent effects (back to front) of metformin.

### 3.2. Metformin Treatment Inhibits Glucose-Stimulated Insulin Secretion

**Figure 2:**
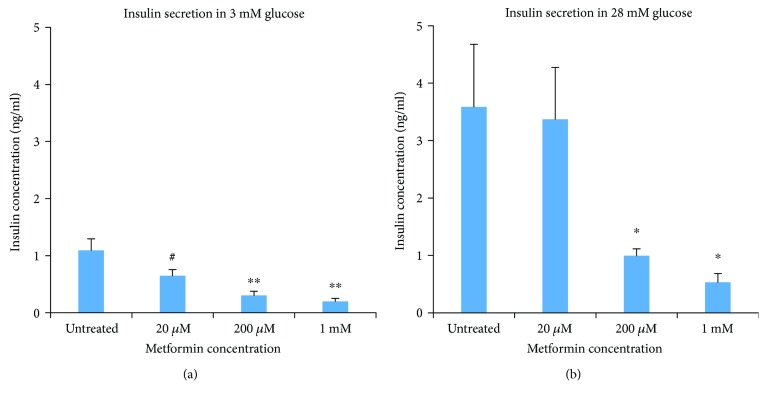
Glucose-stimulated insulin secretion (Mean +/− SEM) is inhibited following 3 days of metformin exposure. Set of 20 islets were incubated in 3 mM glucose (a) or 28 mM glucose (b) following metformin treatment at different concentrations for 3 days. *N* = 5 replicates for each condition conducted in three separate trials using islets from five different mice. #*P* < 0.10, ^∗^*P* < 0.05, ^∗∗^*P* < 0.01.

In a subset of studies, metformin was washed out, and islets were tested for subsequent glucose-stimulated insulin secretion (GSIS) without metformin present. Islets were preincubated in a modified KRB solution containing 0 mM glucose for one hour to minimize glucose metabolism across all treatment conditions (see Methods). Each group was then exposed to 3 mM glucose for 1 h to measure basal insulin secretion in hypoglycemic conditions and then 28 mM glucose for 1 h to measure insulin release in hyperglycemic/diabetic conditions. By making these sequential measurements, we can examine the capacity of islets to respond to a strong glucose (hyperglycemic) stimulus following metformin treatment. Supernatants were collected at the end of each treatment to measure secreted insulin. As shown in Figure 2, significant decreases in insulin secretion with 200 *μ*M and 1 mM metformin were observed for both the 3 mM and 28 mM glucose conditions. Islets in 20 *μ*M metformin showed borderline decreases in insulin secretion in 3 mM glucose (*P* = 0.088), but no difference from untreated controls in 28 mM glucose. It is possible that the effects of 20 *μ*M metformin are rapidly reversible since the effects were not significant.

These observations coincide with the effects of metformin on accumulated insulin in the media and are consistent with at least one previous report [15]. Leclerc et al. showed that metformin at 1 mM concentrations activated the kinase AMP-activated protein kinase (AMPK), which inhibited insulin secretion [15]. Our work suggests that metformin at a 1 mM concentration may be deleterious to islet function and possibly toxic (see below). Importantly, however, we show that metformin decreases insulin secretion at 20 *μ*M concentrations, which is very near the physiologically circulating range of metformin in the body [16–19]. Note that we cannot discount the possibility that metformin may reduce insulin production and/or insulin content since we did not measure this directly.

### 3.3. Metformin May Be Toxic at Higher Concentrations

**Figure 3:**
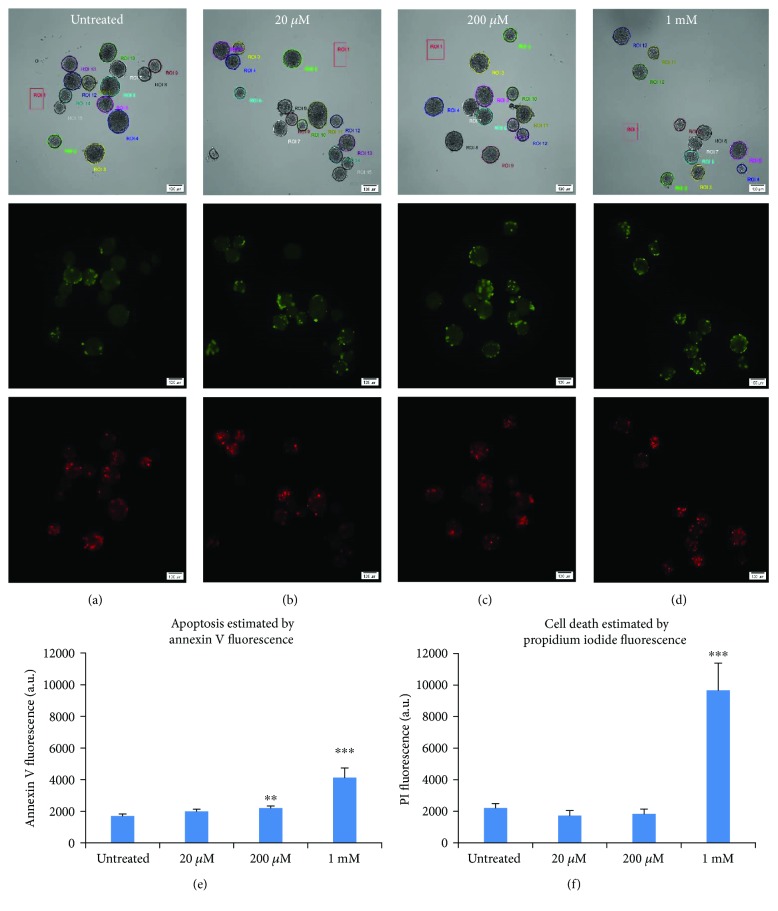
Higher concentrations of metformin induce cell death. (a–d) Images of islets in bright field (top), annexin V fluorescence in green (middle), and PI in red (bottom) for islets exposed to no metformin (a, untreated), 20 *μ*M (b), 200 *μ*M (c), and 1 mM metformin (d). Regions of interest (ROIs) observed in bright field images were drawn around each islet for fluorescence quantification. (e–f) Quantification of fluorescence (Mean +/− SEM) for annexin V (e) and PI (f). ^∗∗^*P* < 0.01, ^∗∗∗^*P* < 0.001. Islet number for each condition ranged from *N* = 19 − 37 from two separate trials.

We also examined islets for cell death following metformin treatment for three days. Islets were imaged for annexin V as a marker of apoptosis and propidium iodide (PI) as an estimate of overall cell death. Islets were imaged in bright field (top), annexin V (middle), and PI (bottom) for panels (a)–(d) with metformin concentrations noted in the top center. Numbered regions of interest around each islet are shown for bright field images. In comparing Figures 3(a)–3(d) by visual inspection, no obvious differences in fluorescence are apparent. When quantified for fluorescence intensity over the surface area of each islet, increased cell death becomes apparent for 1 mM metformin. As shown in Figure 3(e), islets exposed to 20 *μ*M metformin did not show any significant increases in apoptosis, whereas islets exposed to 200 *μ*M showed a small (~30%) but significant increase in apoptosis (*P* < 0.01), and islets exposed to 1 mM showed substantial increases in apoptosis. Cell death as measured by PI was not significantly increased for any condition except of 1 mM metformin (Figure 3(f)). Reduced insulin secretion and islet function thus may be due at least in part to toxic effects of metformin at 1 mM, but this possibility is less likely for 20 and 200 *μ*M.

### 3.4. Metformin Treatment Decreases Glucose-Stimulated [Ca^2+^]_i_ in a Concentration- and Duration-Dependent Manner

**Figure 4:**
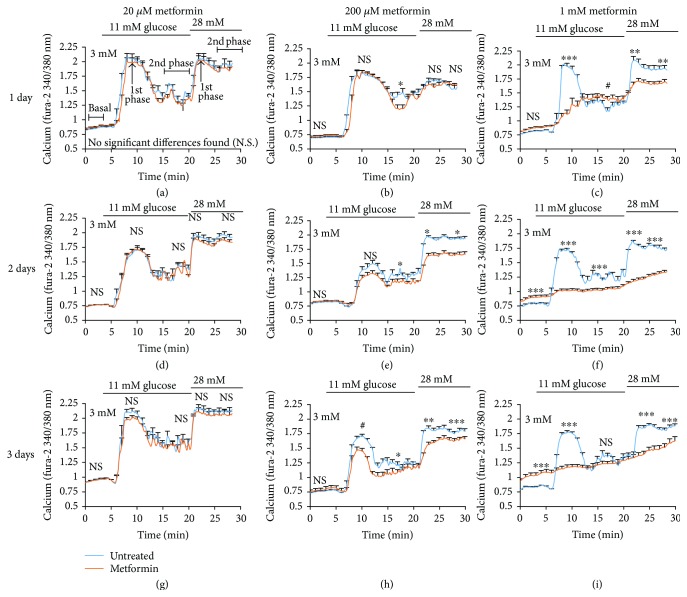
Metformin impairs glucose-stimulated [Ca^2+^]_i_ responses. (a–c) Glucose-stimulated [Ca^2+^]_i_ responses (Mean +/− SEM) from islets treated for 1 day with 20 *μ*M (a), 200 *μ*M (b), and 1 mM metformin (c). (d–f) Glucose-stimulated [Ca^2+^]_i_ responses (Mean +/− SEM) in islets treated for 2 days with 20 *μ*M (d), 200 *μ*M (e), and 1 mM metformin (f). (g–i) Glucose-stimulated [Ca^2+^]_i_ responses (Mean +/− SEM) in islets treated for 3 days with 20 *μ*M (g), 200 *μ*M (h), and 1 mM metformin (i). A total of 436 individual islet traces were studied in two trials combined for analysis; 17–33 islets were recorded for each metformin condition. Phases of the calcium response are labeled only in (a) but occur similarly for each panel. Statistically different means in [Ca^2+^]_i_ between untreated and metformin-treated islets are reported as follows: N.S. = not significant, #*P* < 0.10, ^∗^*P* < 0.05, ^∗∗^*P* < 0.01, ^∗∗∗^*P* < 0.001.

To acquire more detail on the kinetics of metformin's effects on the coupling between glucose stimulation and insulin release, we measured changes in intracellular [Ca^2+^]_i_ (the proximal step to insulin release) at 5-sec intervals in response to acute glucose stimulation. Islets were treated with metformin at 20 *μ*M, 200 *μ*M, and 1 mM concentrations for 1, 2, or 3 days. Fresh media and metformin were given each day. In Figure 4, paired data comparing untreated versus metformin-pretreated islets from one of two trials is displayed. Note that statistical differences shown in Figure 4 were calculated for combined data from both trials and thus may not perfectly reflect what is shown in the traces.

As shown in Figures 4(a)–4(c), following metformin exposure for 1 day, 20 *μ*M metformin had no significant effect on [Ca^2+^]_i_ (Figure 4(a)), 200 *μ*M metformin slightly inhibited the second phase of 11 mM glucose stimulation (Figure 4(b)), but 1 mM metformin nearly abolished the first phase response to 11 mM glucose and significantly impaired the [Ca^2+^]_i_ response to 28 mM glucose (Figure 4(c)). By 2 days of metformin exposure, 20 *μ*M metformin still showed no effect (Figure 4(d)), whereas islets exposed to 200 *μ*M metformin showed the same loss of second phase response to 11 mM glucose and also a marked decrease in the [Ca^2+^]_i_ response to 28 mM glucose (Figure 4(e)). Metformin at 1 mM for 2 days resulted in severe loss of normal islet function (Figure 4(f)). By 3 days, metformin surprisingly showed no significant effect at 20 *μ*M (Figure 4(g)), despite a 40–50% loss in insulin release as shown above (see Figure 1). Exposure to 200 *μ*M appeared to have similar effects at 3 days (Figure 4(h)) as it did at 2 days, which is consistent with the insulin secretion data. Finally, islets exposed to 1 mM metformin for 3 days showed a very elevated basal [Ca^2+^]_i_ (i.e., [Ca^2+^]_i_ in low glucose) and virtually no discernible [Ca^2+^]_i_ response to glucose stimulation (Figure 4(i)).

It should be noted that metformin also appeared to cause disruptions in normal [Ca^2+^]_i_ oscillations, particularly at 1 mM concentrations. Even at 20 *μ*M, there was a significant decline at 3-day exposure in the number of islets displaying oscillations (67% for untreated versus 37% for 20 *μ*M metformin, *P* < 0.05). Because intrinsic oscillations in glycolysis and [Ca^2+^]_i_ are easily disrupted by changes in glucose, islets should be maintained in a steady-state 11 mM glucose condition for proper recording and analysis [29, 30]. For this reason, we chose not to do detailed analysis on this data set.

### 3.5. Dissociations between Metformin-Induced Effects on [Ca^2+^]_i_ and Insulin Secretion

**Figure 5:**
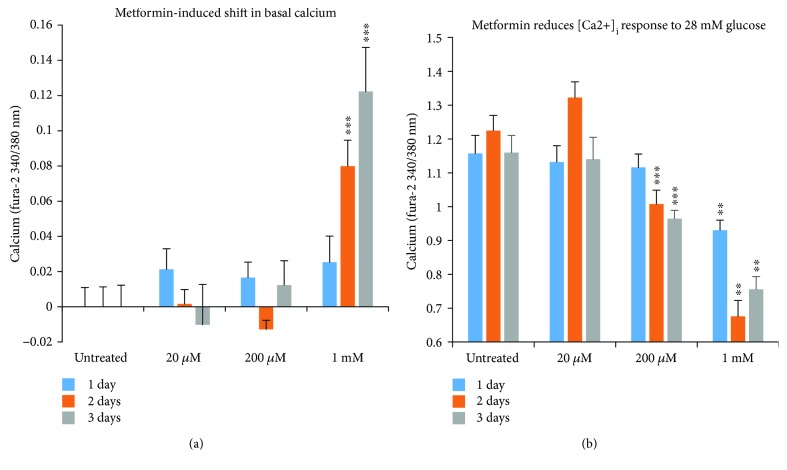
Metformin-induced changes in islet [Ca^2+^]_i_ handling (Mean +/− SEM). (a) Basal shift from control in the fura-2 ratio representing [Ca^2+^]_i_ levels for each concentration and duration of metformin for data described in Figure 4. A large increase in basal [Ca^2+^]_i_ levels (in 3 mM glucose) is seen for islets treated with 1 mM metformin, suggesting possible issues with islet calcium handling. (b) Net increase in fura-2 ratio (peak in 28 mM glucose minus basal) indicating glucose-stimulated change in [Ca^2+^]_i_. ^∗∗^*P* < 0.01, ^∗∗∗^*P* < 0.001.

Analysis of [Ca^2+^]_i_ data for 3 mM glucose and 28 mM glucose are summarized in Figure 5. Under normal conditions, [Ca^2+^]_i_ is tightly regulated and kept low in conditions of low/basal glucose. Issues with basal [Ca^2+^]_i_ are often described as indicators of excessive calcium influx and/or calcium release from the endoplasmic reticulum [24]. As shown in Figure 5(a), significant shifts in basal [Ca^2+^]_i_ relative to control were not found for 20 *μ*M or 200 *μ*M metformin exposure, however, a time-dependent increase in basal [Ca^2+^]_i_ relative to untreated controls was observed for 1 mM metformin. Shifts in fura-2 ratio as little as 0.02 to 0.05 have been associated with islet dysfunction [25, 27]. In the case of the 1 mM concentration, these [Ca^2+^]_i_ responses suggest severe dysfunction similar to the stress-induced effects observed in cytokine-induced cell death [24, 31, 32] or lipotoxicity [27, 33–35]. However, 1 mM metformin is 100x or higher than the typical circulating concentration [16–19], so these effects should not be interpreted as a concern for therapeutic use.

Declines in peak [Ca^2+^]_i_ response to 28 mM glucose stimulation were also concentration- and duration-dependent. Peak [Ca^2+^]_i_ in 28 mM glucose was only slightly reduced for 20 *μ*M, even after 3 days of exposure (and mildly increased at 2 days). This is somewhat incongruous with the insulin secretion data showing ~50% reduction at 3 days but not entirely unexpected since there are well-established dissociations between [Ca^2+^]_i_ and insulin release [27, 36, 37]. The reduction in peak [Ca^2+^]_i_ for 200 *μ*M metformin appears to reflect a concentration-dependent reduction in insulin secretion (Figure 5(b)). Finally, exposure to 1 mM metformin caused a large drop in peak [Ca^2+^]_i_ at each time point (Figure 5(b)). Of interest, the greatest drop occurred for the 2-day exposure with a slightly attenuated drop at 3 days. Given the exceptionally high basal [Ca^2+^]_i_ levels observed at 3 days, the “increase” in peak [Ca^2+^]_i_ may indicate that these islets are no longer able to maintain a proper membrane potential (electrical gradient), as opposed to an improvement in function between 2 days and 3 days of exposure to 1 mM metformin. This is also consistent with the substantial increases in cell death observed for 1 mM metformin.

## 4. Concerns and Limitations

While this work has potential clinical implications, caution must be taken not to overstate these findings. For example, there are numerous anatomical and physiological differences between rodent and human islets which could impact effects of metformin between species [38]. Specifically, human islets have been shown to have low pyruvate carboxylase, ATP citrate lyase, and pyruvate carboxylation but higher acetoacetate compared to rodent islets [39]. These differences in energy metabolism could produce substantial differences in metformin action between rodents and humans. Of note, there have been numerous experiments involving metformin treatment in islets from human donors that were first subjected to hyperglycemic conditions [40, 41], lipotoxic conditions [42, 43], or which originated from donors with T2D [10]. Few studies have reported on the effects of metformin in normal human islets. One report found stimulatory effects of 200 *μ*M metformin in short-term treatment of donor human islets [12], while another study showed inhibitory effects from 200 *μ*M to 1 mM [15]. Thus, concentration and duration of metformin exposure could be as important to explaining different effects as species. We hope to perform future studies to directly examine how metformin impacts the function of human islets.

Throughout this study, we referred to 20 *μ*M metformin as being near the physiological circulating range. We note that while metformin has been reported to peak in the plasma of subjects at ~10 *μ*M [18], average therapeutic levels are typically even lower. Of interest, a database had been created to document circulating metformin levels in patients that were measured for purposes of concentration adjustment, checking for potential metformin accumulation or overdose, or for lactic acidosis [17]. While approximately half of 467 patient samples were found to have plasma metformin levels at or below the therapeutic range (<8 *μ*M), 43% were mildly elevated (up to 30 *μ*M) and 7% of samples were greater than 30 *μ*M [17]. The highest detected plasma metformin level was over 600 *μ*M (113 mg/l). Thus, patients could experience metformin levels in the range of the 20 *μ*M concentration used in our study, but the target therapeutic range for metformin is considerably less than 20 *μ*M.

In addition, the effects of metformin in vivo are complex and multifaceted. Even if the direct effect of metformin on insulin secretion from isolated islets is inhibitory, metformin also impacts other organs that could feedback onto islets. Direct inhibitory effects on islets could be countered by numerous other modulatory factors including reduced hyperglycemia, improved hepatic function, reduced insulin resistance, and so on, all of which would also influence islet function. Thus, the net impact of metformin therapy for patients may not reflect the direct effects of metformin on pancreatic islets in isolation.

## 5. Conclusions

Our work shows that metformin inhibits islet function as measured by insulin release and [Ca^2+^]_i_ changes in response to glucose stimulation. Our findings support other previous studies of metformin's effects on normal islets from mice, rats, and humans [15]. Notably, we show for the first time that (1) metformin impacts calcium handling in islets and (2) metformin can inhibit insulin secretion at concentrations approaching the physiological circulating range.

Metformin's mechanism of action is still under debate. A large amount of evidence points to AMP-activated protein kinase (AMPK) activation as a primary mechanism of action (reviewed in [19]). In beta cells, activation of AMPK would lead to reduced ATP production, which would subsequently increase KATP-channel activity to inhibit insulin secretion. This mechanism would be consistent with the inhibitory effects of metformin we have observed. Alternative mechanisms of action for metformin include inhibition of mitochondrial complex I, which inhibits the mitochondrial respiratory chain [44]. This could also result in reduced oxidative phosphorylation and could also reduce insulin secretion. A number of metabolic intermediates of glucose, amino acids, and fatty acids can also impact insulin secretion [45], and it is possible that metformin could modulate the pathways related to these fuels.

It is possible that the beneficial effect of metformin on islets under various stressors occurs by inhibiting or “resting” fatigued cellular processes. There is evidence showing protective effects of therapies to reduce beta cell workload including intensive insulin therapy at diagnosis with or without metformin, or increasing KATP-channel activity with diazoxide to reduce beta cell excitability [46, 47]. We recently showed that reducing glycolytic activity in islets from diabetic mice can restore normal islet glucose sensing and improve oscillatory function [28].

Overall, our findings indicate that the direct effects of metformin on insulin secretion from islets in isolation are inhibitory. If also true in vivo, our data suggest metformin may benefit individuals with islets that are fatigued by elevated metabolic demands by reducing islet metabolic activity. However, individuals with normally functioning islets may see reduced insulin secretion from metformin treatment. These islet effects are likely secondary to the insulin-sensitizing effects of metformin since the overwhelming clinical evidence indicates that metformin has beneficial metabolic effects overall.
